# Chronic pain and problematic substance use for veterans during COVID-19: the moderating role of psychological flexibility

**DOI:** 10.3389/fpsyg.2023.1173641

**Published:** 2023-05-02

**Authors:** Erin D. Reilly, Noah R. Wolkowicz, Alicia Heapy, R. Ross MacLean, Brooke A. Duarte, Elizabeth S. Chamberlin, J. Irene Harris, Steven D. Shirk, Megan M. Kelly

**Affiliations:** ^1^Mental Illness Research, Education, and Clinical Center (MIRECC), VA Bedford Healthcare System, Bedford, MA, United States; ^2^University of Massachusetts Medical School, Worcester, MA, United States; ^3^Mental Illness Research, Education, and Clinical Center (MIRECC), VA Connecticut Healthcare System, West Haven, CT, United States; ^4^Yale School of Medicine, New Haven, CT, United States; ^5^VA Connecticut Healthcare System Pain Research, Informatics, Multimorbidities, and Education (PRIME) Health Services Research and Development Center of Innovation, West Haven, CT, United States; ^6^Suffolk University, Boston, MA, United States; ^7^University of Minnesota Medical School, Minneapolis, MN, United States

**Keywords:** chronic pain, substance use, resilience, veterans, pandemic, mental health

## Abstract

**Background:**

Chronic pain and problematic substance use are commonly co-occurring and highly detrimental issues that are especially prevalent in U.S. veteran populations. Although COVID-19 made clinical management of these conditions potentially difficult, some research suggests that certain veterans with these conditions did not experience this period as negatively as others. It is thus important to consider whether resilience factors, such as the increasingly-studied process of psychological flexibility, might have led to better outcomes for veterans managing pain and problematic substance use during this time of global crisis.

**Methods:**

This planned sub-analysis of a larger cross-sectional, anonymous, and nationally-distributed survey (*N* = 409) was collected during the first year of the COVID-19 pandemic. Veteran participants completed a short screener and battery of online surveys assessing pain severity and interference, substance use, psychological flexibility, mental health functioning, and pandemic-related quality of life.

**Results:**

For veterans with chronic pain and problematic substance use, the pandemic resulted in a significant lowering of their quality of life related to meeting basic needs, emotional health, and physical health compared to veterans with problematic substance use but no chronic pain diagnosis. However, moderation analyses revealed that veterans with these comorbid conditions experienced less negative impacts from the pandemic on quality of life and mental health when they reported greater psychological flexibility. For veterans with problematic substance use only, psychological flexibility was also related to better mental health functioning, but did not significantly correlate with their quality of life.

**Conclusion:**

Results highlight how COVID-19 differentially impacted veterans with both problematic substance use and chronic pain, such that this group reported particularly negative impacts of the pandemic on multiple areas of quality of life. However, our findings further emphasize that psychological flexibility, a modifiable resiliency process, also buffered against some of the negative impacts of the pandemic on mental health and quality of life. Given this, future research into the impact of natural crises and healthcare management should investigate how psychological flexibility can be targeted to help increase resiliency for veterans with chronic pain and problematic substance use.

## 1. Introduction

Chronic pain and problematic substance use are two frequently co-occurring and significant health problems in the United States. On its own, chronic pain (e.g., pain lasting longer than 3–6 months and persisting beyond the healing of an initial injury or disease) is very complicated to treat (Qaseem et al., 2017; Dowell et al., 2022) and associated with a multitude of negative health problems and functional issues (Kerns et al., 2003; Rice et al., 2016; Hadi et al., 2019). In an effort to alleviate pain, many individuals turn to substances such as alcohol, tobacco, and opioids (Blanco et al., 2016; Ditre et al., 2019), leading to a higher prevalence of comorbid chronic pain and substance use concerns (Manhapra and Becker, 2018). The resulting interaction between chronic pain and substance use is complex. For instance, although frequent use of substances can have short-term analgesic impacts, pain is often exacerbated during subsequent abstinence periods (Jochum et al., 2010; Ditre et al., 2018), and repeated opioid misuse can also induce hyperalgesia (e.g., increased pain sensitivity; Benyamin et al., 2008; Compton et al., 2012; Hooten et al., 2015). When chronic pain prompts self-medication through substances, this in turn contributes to escalating problematic substance use and poorer pain-treatment outcomes, resulting in a positive feedback loop maintaining both issues (Ditre et al., 2019). Even in the absence of a formal substance use diagnosis, individuals with chronic pain can experience greater levels of pain due to problematic substance use or drinking and drug use that does not meet full substance use disorder criteria but is linked to personal distress, physical health issues, or legal or social concerns (e.g., Alford et al., 2016; Bello et al., 2018).

Comorbid chronic pain and problematic substance use are especially prevalent in veteran populations (Oliva et al., 2017; Hoggatt et al., 2021). Compared to their civilian adult counterparts, veterans regularly demonstrate a higher prevalence of both chronic pain (e.g., past 6-month prevalence: 29.1% vs. 19.5%; Dahlhamer et al., 2018) and substance use disorders (past 12-month prevalence: 12.8%; Hoggatt et al., 2021 vs. 3.0% National Survey on Drug Use and Health (NSDUH), 2019). Veterans with chronic pain are also at an increased risk for developing substance use issues (Morasco et al., 2011a). This comorbid chronic pain and substance misuse, including prescribed opioid misuse as well as other substances such as tobacco and alcohol, can result in multiple severe negative impacts such as mental and physical health issues (Ward et al., 2022), difficulties maintaining employment (Wyse et al., 2021), and lack of stable housing (Dunne et al., 2015).

Veterans with both chronic pain and problematic substance use may have found it particularly challenging to effectively manage their health and well-being during the COVID-19 pandemic. Many patients with chronic pain, including those treated with prescribed opioids, faced cancelled or postponed pain treatment due to the pandemic (Eccleston et al., 2020) even though research has shown that COVID-19 prompted worsening pain, depression symptoms, stress, social isolation, and decreased quality of life (e.g., Purcell et al., 2021; Compton and Marie, 2022). At the same time, studies conducted during the pandemic also identified resilient subsets of veterans who maintained high functioning despite their pain symptoms. For example, Lazar et al. (2022) found that despite COVID-related disruptions in care, many veterans with chronic pain saw the pandemic as an opportunity to enact positive lifestyle changes (e.g., working on work-life balance, joining a community bike ride) that increased their quality of life. Qualitative studies have also reported positive changes for veterans during COVID-19, such as allowing them more time at home to focus on beneficial health behaviors and re-engage with family members (Mohamed Ali et al., 2022).

Understanding what factors may have helped veterans adopt positive behaviors to improve their pain experience and quality of life during COVID-19 could help inform future pain and substance use treatment. One such protective factor shown to buffer against negative mental health and functional impacts for individuals facing challenging life circumstances is psychological flexibility (Gentili et al., 2019; Meyer et al., 2019). Psychological flexibility is characterized by an ability to remain present-focused and fully aware of emotions, sensations, and thoughts, be willing to accept them, and choose to act in ways that align with individual values (Hayes et al., 2004). Psychological flexibility fundamentally reflects how an individual adapts to difficult situations and stressful life events and how they cope with their internal experiences in those moments (Kashdan and Rottenberg, 2010). Such characteristics are, in turn, associated with improved mental health functioning and quality of life (Block and Block, 2006; Smeekens et al., 2007; Rüsch et al., 2008; Berking et al., 2009) and have led many researchers to view it as a transdiagnostic protective factor (e.g., Kashdan and Rottenberg, 2010; Levin et al., 2014; Kroska et al., 2020; Pakenham et al., 2020).

The protective qualities of psychological flexibility may be of particular importance when considering the coping of individuals with chronic pain and problematic substance use (e.g., Li et al., 2019; Gloster et al., 2021; Mallik et al., 2021). Notably, psychological flexibility supports self-efficacy for both pain and addiction management by increasing perceived power to enact positive change in one’s life and promoting values-oriented, goal-directed action (Hayes et al., 2004). Understanding the benefit of increased psychological flexibility during this time may also be key to assisting these veterans, as psychological flexibility is a potentially modifiable factor through therapeutic interventions such as Acceptance and Commitment Therapy (ACT) and other mindfulness and acceptance-based interventions (Rash et al., 2019). Among individuals with chronic pain, psychological flexibility has been associated with less pain-related physical impairment (Vowles and McCracken, 2008), increased quality of life (Duarte et al., 2022), and reduced substance misuse (Gentili et al., 2019; Li et al., 2019). Thus, psychological flexibility may be an important factor in understanding how veterans with both chronic pain and substance use concerns managed the COVID-19 pandemic and can continue to manage symptoms in the everchanging public health landscape.

### 1.1. The current study

The present study examined the relationship between chronic pain and psychological flexibility on mental health and pandemic-related functioning for veterans reporting problematic substance use. Advancing our understanding of factors that can buffer against pandemic-induced deteriorations in functioning is especially important for promoting the well-being of veterans with co-occurring chronic pain and substance use concerns. We investigated the following hypotheses:

*Hypothesis 1*: Veterans reporting problematic substance use and a prior chronic pain diagnosis (vs. no chronic pain diagnosis) would report greater perceived deterioration in major areas for quality of life (i.e., finances, ability to meet basic needs, emotional health, concentration, and physical health).

*Hypothesis 2*: That psychological flexibility would be positively correlated with mental health functioning, and negatively correlated with problematic substance use, self-reported pain, and pandemic-related worsening of quality of life for all veterans.

*Hypothesis 3*: That psychological flexibility would moderate the relationship between chronic pain diagnosis and mental health functioning, such that lower levels of psychological flexibility will be associated with significantly poorer mental health functioning for veterans with chronic pain and problematic substance use. In addition, at higher levels of psychological flexibility, all veterans (i.e., with and without chronic pain diagnosis) would report greater mental health functioning.

*Hypothesis 4*: That the same interaction pattern proposed in Hypothesis 3 between psychological flexibility and chronic pain diagnosis would be found for self-reported pandemic-related quality of life, such that greater psychological flexibility would be associated with better quality of life during COVID-19 for veterans with chronic pain and problematic substance use. We also hypothesized that, at higher levels of psychological flexibility, all veterans would report less negative impact of COVID-19 on quality of life.

## 2. Materials and methods

### 2.1. Participants and procedure

This study was an *a priori* planned sub-analysis of an online survey of the effect of COVID-19 on physical and mental health of veterans with self-reported problematic drug and alcohol use administered using the Qualtrics federal platform between November 24, 2020, and February 2, 2021 (results of the primary study published elsewhere: Reilly et al., 2022). Procedures and methods for collecting this data were approved by the Institutional Review Board at the VA Bedford Healthcare System. Survey participants were recruited and identified through the Qualtrics federally-approved survey platform using their panel-aggregator system. Participants had previously signed-up for specific online survey panels available through Qualtrics and were provided *via* the panel aggregator system with basic information about the survey and a hyperlink. Subsequently, potential participants provided informed consent and then completed items assessing inclusion/exclusion criteria. Eligible participants were at least 18 years old and reported being a U.S. military veteran, which was confirmed through the review of multiple items related to age, service years, and DD214 information. Eligible participants reported problematic substance use as defined by a minimum score of 1 on the CAGE Adapted to Include Drugs (CAGE-AID; Brown and Rounds, 1995). This procedure has been recommended by the Consensus Panel for sufficient breadth in identifying veterans with potential SUDs (Sullivan and Fleming, 2008). Participants were then provided access to the survey, which was estimated to take approximately 25 min to complete. Respondents were paid $4.80 to complete the survey.

A total of 436 participants completed the survey. Twenty-seven respondents (6.2%) were removed for not meeting pre-determined quality review standards (see primary data Reilly et al., 2022 for more information on the quality review process). The final data set consisted of 409 participants. Across the full sample, most respondents were male (76.5%), not Hispanic/Latino (91.9%), heterosexual (91.4%), and served after 1990 (61.6%), with a mean age of 54.96 (SD = 16.44). Similar to prior studies (Dahlhamer et al., 2018), a total of 31.5% (129/409) of participants self-reported receiving a chronic pain diagnosis from a health provider.

### 2.2. Study measures

Survey-collected demographics included self-reported age, gender, sexual orientation, race, ethnicity, income, armed service era, use of VHA services, and relationship status. Participants also self-reported whether they had received a diagnosis of chronic pain or substance use disorder (defined for survey participants as a formal diagnosis received from a doctor or healthcare provider). Participants’ self-reported diagnosis of chronic pain was used to group respondents into chronic and non-chronic pain categories for analysis.

The three-item Pain, Enjoyment, General Activity scale (PEG-3) was used to assess (1) pain intensity, (2) pain-related interference with enjoyment of life, and (3) pain-related interference with daily activities over the prior week (Krebs et al., 2009). The PEG-3 has been found to be sensitive to change and differentiate between individuals with and without pain improvement over time (Krebs et al., 2010). The PEG-3 items differentiate levels of pain severity continuously from a zero to ten scale. The composite PEG score, created by summing all items and dividing by three, can be viewed as a measure of total pain impact. For the current sample, internal consistency was high for the PEG-3 (*α* = 0.90).

The Cut Down, Annoyed, Guilty, Eye Opener (CAGE) Adapted to Include Drugs (CAGE-AID; Brown and Rounds, 1995) was used to screen for problematic substance use. This is a validated 4-item measure that assesses the impact of participants’ use of alcohol and other drugs and its severity. Per the CAGE-AID protocol, the questionnaire was only given to participants who reported current alcohol or drug use. It consists of four yes/no questions, “Have you ever felt you ought to cut down on your drinking or drug use,” “Have people annoyed you by criticizing your drinking or drug use,” “Have you ever felt bad or guilty about your drinking or drug use,” and “Have you ever had a drink or used drugs first thing in the morning to steady your nerves or to get rid of a hangover.” The CAGE-AID has demonstrated both high internal consistency and high sensitivity and specificity to screen for problematic substance use (McIntyre et al., 2021). It is not intended as a diagnostic tool.

Psychological flexibility was measured using the Psyflex Measure (Gloster et al., 2021), which assesses the major aspects of psychological flexibility: acceptance, mindfulness, and values-oriented actions in daily living. Items are rated on a Likert scale from 1 = very seldom to 5 = very often and summed. The six-item measure aims to measure psychological flexibility by capturing each domain of the ACT Hexaflex (Hayes et al., 1999, 2011) including present moment-centeredness, acceptance, cognitive defusion, self-as-context, values, and committed action. Higher scores represent higher psychological flexibility. According to Constantinou et al. (2021), the Psyflex supported a one-factor solution across four clinical and non-clinical samples and demonstrated good psychometric properties (Cronbach’s alpha = 0.81; Gloster et al., 2021). For the current study, the Psyflex was found to be highly reliable (*α* = 0.88).

The Pain Management Collaboratory Coronavirus Pandemic COVID-19 5-Item Measure (PMC-5; Coleman et al., 2021) was created early in the pandemic to assess the potential negative impact of COVID-19 on quality of life. The developers of this measure suggest individualized adaptation as appropriate of this scale by clinical research teams to survey the effects of the COVID-19 pandemic on study participants, leading to the inclusion of three of the original items five domains of pandemic impact in the current study: finances, ability to meet basic needs, and emotional health. Two additional areas were added to the scale, physical health and ability to concentrate, to assess physical and cognitive functioning changes, respectively. These domains were chosen based on nascent COVID-19 literature suggesting these social determinants of health management—financial, physical, and emotional - might be particularly impacted during the pandemic (e.g., Ryan et al., 2020). Each quality of life domain was assessed on a Likert scale from 1 = improved to 4 = a lot worse. Items were assessed both individually and as a total score reflecting pandemic-related quality of life for veterans. In the current sample, internal validity was satisfactory (Cronbach’s alpha = 0.82).

Mental health functioning was measured using the Short-Form Health Survey-12 (SF-12; Ware et al., 1996), a 12-item measure that assesses global areas functioning. The SF-12 has been validated for predicting populations’ mental health and functioning without targeting specific health outcomes and has high reliability, including with U.S. veterans (Salyers et al., 2000). The current study utilized only the mental health composite score (MCS-12) of the SF-12 as a measure of global mental health functioning, in line with past research using the SF-12 subscale specifically for mental health measurement (e.g., McAlpine et al., 2018). Scoring involves using a norm-based algorithm that produces a self-reported MCS-12 between 0 and 100 (Jones et al., 2001), with lower scores associated with lower mental health functioning. Previous studies have indicated acceptable 2-week test–retest reliability of 0.76 for the MCS-12 in the general U.S. population (Ware et al., 1996).

### 2.3. Data analysis

Initial descriptive statistics were examined using frequencies (n) and percentages (%), with independent sample t-tests and Chi-square tests used to assess for potential differences in sociodemographic variables (e.g., age, gender, use of VHA services), average pain level (PEG-3), and problematic substance (CAGE-AID) use by chronic pain diagnosis status. Statistical analysis for Hypothesis 1 then consisted of a comparative analysis of participants with and without a self-reported diagnosis of chronic pain on quality of life using independent samples t-tests for each categorical variable on the PMC-5 (i.e., finances, basic needs, concentration and focus, mental health, and physical health). To analyze initial relationships between variables of interest, Pearson Product correlations were conducted for continuous variables to investigate indications of multicollinearity in the planned hierarchical multiple regression analysis for Hypotheses 3 and 4. Multicollinearity among predictor variables was set at zero-order correlations greater than 0.70, and continuous variables scores and errors were inspected for normalcy of distribution and residuals. Continuous predictor variables were mean-centered prior to computing an interaction term for the regression model. Hierarchical linear regression was utilized to investigate Hypothesis 3, the relationship between chronic pain diagnosis and mental health functioning, as potentially moderated by psychological flexibility, and Hypothesis 4, the relationship between chronic pain diagnosis and pandemic-related quality of life as moderated by psychological flexibility. To correct for multiple tests, a Bonferroni adjusted alpha level of 0.05/k, where k was set at 6 (the number of predictors in the model) was used and partial regression coefficients were set to an adjusted alpha of 0.008. Survey participants were required to answer all survey questions, and thus the data set included no missing data.

Preliminary data analyses demonstrated that key assumptions for multiple regression analysis were met. Simple slopes analyses were conducted for significant interactions. In the first step, regression estimates controlled for age, self-reported problematic substance use behaviors, and total pain impact. In the second step, hypothesized predictors of psychological flexibility and chronic pain diagnosis were added to the model to assess for main effects. In the third step, the cross-product of psychological flexibility and chronic pain diagnosis was added to evaluate for the predicted interaction. These steps were repeated to test the same interaction model for Hypothesis 4 with the dependent variable of pandemic-related quality of life. All analyses were performed using IBM SPSS v26.

## 3. Results

### 3.1. Chronic pain diagnosis and pandemic-related quality of life

For respondents with a self-reported chronic pain diagnosis, the mean composite PEG-3 score over the prior week was 5.95 (SD = 2.29), which was significantly higher than those not reporting a chronic pain diagnosis (M = 3.30, SD = 2.64), *t*(407) = −2.81, *p* < 0.001, *d* = −1.04. A Chi-squared test revealed that compared to participants with problematic substance use and no chronic pain diagnosis, participants with both substance use concerns and a chronic pain diagnosis were more likely to have received clinical care from the VA, χ2 (1, N = 409) = 13.60, *p* < 0.01 and identify as a race other than Caucasian, *χ*^2^ (1, N = 409) = 5.89, *p* = 0.02. Veterans with or without chronic pain were equally likely to self-report having a formal substance use disorder diagnosis. Sociodemographic characteristics are presented for the full sample and by chronic pain diagnosis in Table 1.

**Table 1:** Sample demographics by chronic pain diagnosis category (N = 409).

Variable	All respondents	Chronic pain diagnosis	No chronic pain diagnosis
	*N* = 409	*n* = 129	*n* = 290
**Gender**			
Male	313 (76.5%)	92 (71.3%)	221 (78.9%)
Female	94 (23%)	36 (27.9%)	58 (20.7%)
Transgender Male	1 (0.2%)	0 (0.0%)	1 (0.4%)
Preferred not to answer	1 (0.2%)	1 (0.8%)	0 (0.0%)
Age (M, SD)	54.96 (16.44)	54.96 (15.09)	54.80 (17.05)
CAGE-AID	2.37 (1.12)	2.48 (1.17)	2.31 (1.10)
PEG-3**	4.14 (2.82)	5.94 (2.29)	3.30 (2.64)
Race*			
White	370 (90.5%)	110 (85.3%)	260 (92.9%)
Black/African American	22 (5.4%)	10 (7.8%)	12 (4.3%)
Other	7 (1.7%)	2 (1.6%)	5 (1.8%)
Asian	5 (1.2%)	3 (2.3%)	2 (0.7%)
Native Hawaiian/Pacific Islander	5 (1.2%)	4 (3.1%)	1 (0.4%)
American Indian/Alaska Native	5 (1.2%)	1 (0.8%)	4 (1.4%)
**Ethnicity**			
Not Hispanic/Latino	376 (91.9%)	117 (90.7%)	259 (92.5%)
Hispanic/Latino	33 (8.1%)	12 (9.3%)	21 (7.5%)
Sexual orientation			
Heterosexual (straight)	374 (91.4%)	120 (93%)	254 (90.7%)
Bisexual	19 (4.6%)	7 (5.4%)	12 (4.3%)
Gay/lesbian	13 (3.2%)	2 (1.6%)	11 (3.9%)
Prefer not to say	3 (0.7%)	0 (0.0%)	3 (1.1%)
**Service era**			
September 2001 or later	156 (38.1%)	48 (37.2%)	108 (38.6%)
August 1990 to August 2001	96 (23.5%)	39 (30.2%)	57 (20.4%)
May 1975 to July 1990	99 (24.2%)	41 (31.8%)	58 (20.7%)
Vietnam Era (1964–1975)	150 (36.7%)	46 (35.7%)	104 (37.1%)
February 1955 to July 1964	24 (5.9%)	3 (2.3%)	21 (7.5%)
Korean War (1950 to 1955)	3 (0.7%)	1 (0.8%)	2 (0.7%)
**Income**			
Less than $19,999	31 (7.60%)	13 (10.1%)	18 (6.4%)
$20,000–$39,999	70 (17.11%)	25 (19.4%)	45 (16.1%)
$40,000–$59,999	66 (16.14%)	25 (19.4%)	41 (14.6%)
$60,000–$79,999	53 (12.96%)	19 (14.8%)	34 (12.1%)
$80,000–$99,999	48 (11.74%)	14 (10.9%)	34 (12.1%)
$100,000–$149,999	87 (21.30%)	18 (14.0%)	69 (24.6%)
$150,000 +	54 (13.20%)	15 (11.6%)	39 (13.9%)
Comorbid substance use disorder	84 (20.5%)	28 (21.0%)	56 (19.3%)
Received VHA care**	268 (65.5%)	101 (78.3%)	167 (59.6%)

Participants could choose multiple race and service era categories.

Differences between diagnostic groups denoted with *p < 0.05; **p < 0.001.

**Figure 1:**
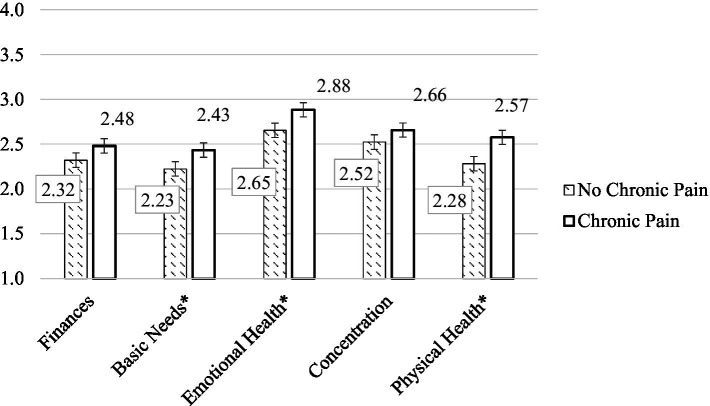
Decline in major areas of quality of life during COVID-19. *indicates significant difference between groups.

To test Hypothesis 1, independent-samples t-tests were conducted to compare veterans reporting problematic substance use with and without comorbid chronic pain across five self-reported areas of quality of life measured by the PMC-5, including finances, ability to meet basic needs, emotional health, concentration, and physical health (see Figure 1). There was a significant difference in the scores for perceived worsening of ability to meet basic needs by diagnosis for veterans with chronic pain and problematic substance use (*M* = 2.43, SD = 0.76) and those with problematic substance use but without chronic pain (*M* = 2.22, SD = 0.67) conditions; *t*(407) = −2.81, *p* = 0.008, *d* = −0.30; self-reported emotional health for veterans with chronic pain (*M* = 2.88, SD = 0.85) and without chronic pain (*M* = 2.65, SD = 0.75) conditions; *t*(406) = −2.63, *p* = 0.006, *d* = −0.30; and perceived physical health for veterans with chronic pain (*M* = 2.28, SD = 0.66) and without chronic pain (*M* = 2.57, SD = 0.79) conditions; *t*(404) = −3.61, *p* < 0.001, *d* = −0.41. No significant differences were found for finances (*p* = 0.08) or ability to concentrate and focus (*p* = 0.12).

### 3.2. Regression analyses

Preliminary comparison tests for planned regression analyses were conducted to investigate possible differences by primary variables of interest (chronic pain diagnosis, mental health functioning, and psychological flexibility), potential control variables (overall pain score, problematic substance use), and demographics (age) on outcome variables. Younger age, higher levels of problematic substance use (CAGE-AID), and greater self-reported pain (PEG-3) were significantly related to poorer mental health functioning (MCS-12) and greater pandemic-related stressors (PMC-5; see Table 2). Consequently, age, CAGE-AID, and PEG-3 were controlled for when conducting the subsequent hierarchical multiple regression to explain the variance in mental health and pandemic-related functioning. Additionally, correlation analyses (see Table 2) were reviewed to assess Hypothesis 2. Mental health functioning was negatively correlated with pain and substance use concerns, and positively correlated with psychological flexibility for veterans with and without a formal chronic pain diagnosis. All continuous variables had an approximate normal distribution with no significant outliers, and statistically significant relationships among variables did not suggest multicollinearity (absolute correlation coefficient greater than 0.70).

**Table 2:** Correlations among continuous predictor variables.

Measure	1	2	3	4	5	6
1. Age	–					
2. PEG-3	−0.30***	–				
3. CAGE-AID	−0.33***	0.26***	–			
4. Psyflex	0.21***	−0.12*	−0.14**	–		
5. MCS-12	0.28***	−0.16**	−0.24***	0.35***	–	
6. PMC-5	−0.31***	0.34***	0.31***	−0.30***	−0.33***	
*M*	54.84	4.14	2.37	38.75	42.43	2.47
*SD*	16.44	2.82	1.12	8.56	6.78	0.57
Range	19–88	0–10	1–4	11–55	20–64	1–4

PEG-3, The Pain, Enjoyment of Life and General Activity Scale; CAGE-AID, The CAGE Adapted to Include Drugs Questionnaire; Psyflex, Psychological flexibility scale; MCS-12, Short-Form 12 Health Survey Mental Component Score; PMC-5, Pandemic-Related Quality of Life.

*p < 0.05; **p < 0.01; ***p < 0.001.

Hierarchical multiple regression was used to investigate Hypothesis 3, that the relationship between chronic pain and mental health functioning during COVID-19 would be moderated by an individual’s self-reported psychological flexibility (see Table 3). Preliminary data analyses were conducted and assured that key assumptions for multiple regression analysis were met. The interaction between chronic pain and psychological flexibility emerged as a significant predictor of mental health functioning (*β* = 0.15, SE = 0.07, *p* = 0.007).

**Table 3:** Hierarchical multiple regression testing interaction model on mental health.

Step and predictors	Mental health functioning					
	Δ*R*^2^	*β*	*B*	SE	sr2	95% CI for *B*
Step 1	0.11					
Age		0.22***	0.09	0.02	0.20	[0.05, 0.13]
CAGE-AID		−0.16**	−0.93	0.31	−0.14	[−1.53, −0.33]
PEG-3		−0.05	−0.12	0.12	−0.05	[−0.36, 0.12]
Step 2	0.09					
Age		0.18***	0.18	0.02	0.16	[0.03, 0.11]
CAGE-AID		−0.14**	−0.14	0.29	−0.13	[−1.39, −0.25]
PEG-3		0.01	0.01	0.13	0.00	[−0.24, 0.27]
Psyflex		0.29***	0.29	0.04	0.29	[0.16, 0.30]
Chronic Pain Diagnosis		−0.08	−0.08	0.74	−0.07	[−2.61, 0.28]
Step 3	0.01					
Age		0.17***	0.07	0.02	0.16	[0.03, 0.11]
CAGE-AID		−0.14**	−0.83	0.29	−0.13	[−1.39, −0.26]
PEG-3		−0.01	−0.02	0.13	−0.01	[−0.27, 0.23]
Psyflex		0.20***	0.16	0.05	0.16	[0.07, 0.25]
Chronic Pain Diagnosis		−0.08	−1.10	0.73	−0.07	[−2.55, 0.32]
Psyflex X Chronic Pain		0.15**	0.20	0.07	0.12	[0.05, 0.35]

PEG-3, The Pain, Enjoyment of Life and General Activity Scale; CAGE-AID, The CAGE Adapted to Include Drugs Questionnaire; Psyflex, Psychological flexibility scale.

*p < 0.05, **p < 0.01, ***p < 0.001.

**Figure 2:**
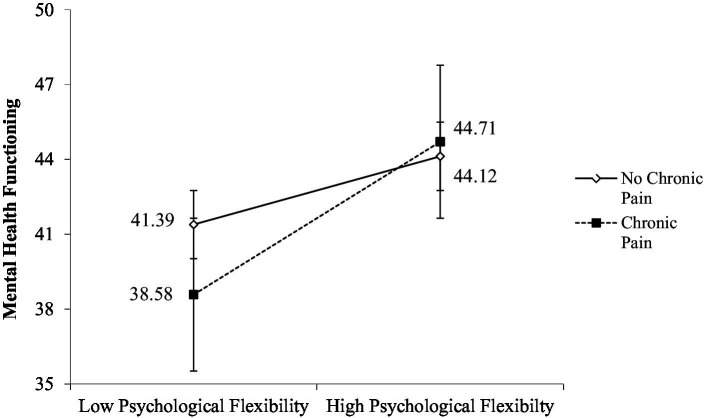
Interaction of psychological flexibility and chronic pain on mental health functioning.

Simple slopes analysis was consequently conducted to probe the interaction. Findings indicate that for veterans without chronic pain, higher levels (1 SD above the mean) of psychological flexibility were associated with greater mental health functioning, *B* = 0.16, *β* = 0.20, SE = 0.05, *p* < 0.001 (see Figure 2). This relationship was even stronger for veterans with chronic pain, such that those who reported greater psychological flexibility also reported better mental health functioning, *B* = 0.36, *β* = 0.45, SE = 0.06, *p* < 0.001. This indicates that psychological flexibility was a predictor for greater mental health functioning for veterans with substance use concerns, with or without chronic pain—though the veterans with chronic pain showed particularly positive mental health impacts related to greater psychological flexibility. The overall model explained 21% of the variance in mental health functioning, total *R*^2^ = 0.21, *F*(6, 402) 17.34, *p* < 001.

**Figure 3:**
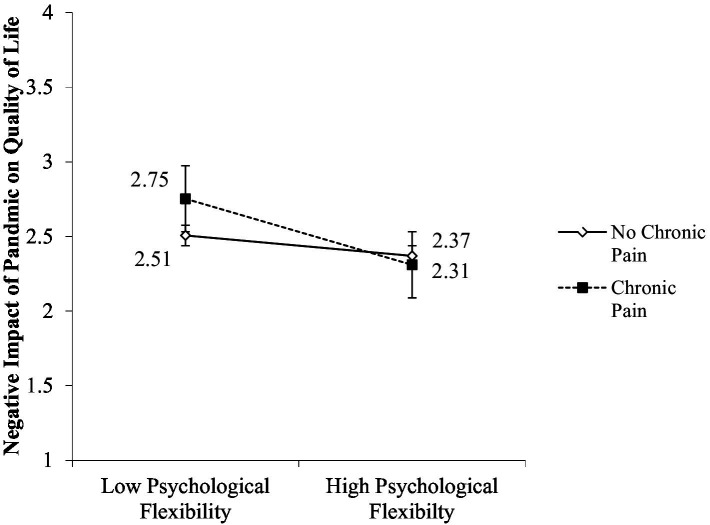
Interaction of psychological flexibility and chronic pain on pandemic-related quality of life.

Per Hypothesis 4, we also expected the pattern of interaction found for mental health functioning would hold for pandemic-related quality of life (Table 4). The interaction between chronic pain and psychological flexibility emerged as a significant predictor of pandemic-related quality of life (*β* = −0.16, *p* = 0.003). Simple slopes analysis revealed that for veterans without chronic pain, higher levels (1 SD above the mean) of psychological flexibility was not significantly associated with better pandemic-related quality of life, *B* = −0.01, *β* = 0.12, SE = 0.00, *p* = 0.03 (see Figure 3). This relationship was, however, significant for veterans with chronic pain, such that those reporting greater psychological flexibility also reported decreased negative impacts to quality of life due to the pandemic, *B* = −0.03, *β* = 0.39, SE = 0.00, *p* < 0.001. This indicates that psychological flexibility was a significant predictor for pandemic-related quality of life, with veterans with chronic pain showing less perceived negative impact of the pandemic on their quality of life when they reported greater psychological flexibility. The overall model explained 26% of the variance in mental health functioning, total *R*^2^ = 0.26, *F*(6, 402) = 23.73, *p* < 001.

**Table 4:** Hierarchical multiple regression testing interaction model on pandemic-related quality of life.

Step and predictors	Pandemic-related quality of life					
	Δ*R*^2^	*β*	*B*	SE	sr2	95% CI for *B*
Step 1	0.20					
Age		−0.18***	−0.01	0.00	−0.17	[−0.01, −0.01]
CAGE-AID		0.18***	0.09	0.02	0.17	[0.05, 0.14]
PEG-3		0.24***	0.05	0.01	0.23	[0.03, 0.07]
Step 2	0.05					
Age		−0.16**	−0.01	0.00	−0.14	[−0.01, −0.01]
CAGE-AID		0.17***	0.09	0.02	0.16	[0.04, 0.13]
PEG-3		0.19***	0.04	0.01	0.16	[0.02, 0.06]
Psyflex		−0.22***	−0.02	0.00	−0.24	[−0.02, −0.01]
Chronic Pain Diagnosis		0.08	0.10	0.06	0.08	[−0.02, 0.22]
Step 3	0.02					
Age		−0.15**	−0.01	0.00	−0.17	[−0.01, −0.01]
CAGE-AID		0.17***	0.09	0.02	0.18	[0.04, 0.13]
PEG-3		0.21***	0.04	0.01	0.18	[0.02, 0.06]
Psyflex		−0.12*	−0.01	0.00	−0.23	[−0.02, −0.01]
Chronic Pain Diagnosis		0.08	0.09	0.06	0.07	[−0.03, 0.20]
Psyflex X Chronic Pain		−0.16**	−0.02	0.01	−0.13	[−0.02, −0.01]

PEG-3, The Pain, Enjoyment of Life and General Activity Scale; CAGE-AID, The CAGE Adapted to Include Drugs Questionnaire; Psyflex, Psychological flexibility scale.

*p < 0.05, **p < 0.01, ***p < 0.001.

## 4. Discussion

The current cross-sectional study of veterans with problematic substance use found that psychological flexibility appeared to buffer negative outcomes in mental health functioning and pandemic-related quality of life among veterans with co-occurring chronic pain. This buffering effect was also present among veterans with only problematic substance use (but without chronic pain) in relation to their mental health functioning, but not for their pandemic-related quality of life. Finally, although both groups reported decrements in quality of life due to the pandemic, those with comorbid chronic pain and problematic substance use reported reduced functioning across multiple discrete areas of quality of life that they specifically attributed to the pandemic, including meeting basic needs, emotional health, and physical health relative to those without chronic pain.

Our finding of the moderating effect of psychological flexibility on well-being aligns with international research suggesting that psychological flexibility may have served as a potential resiliency factor for adults during the first wave of COVID-19 (McCracken et al., 2021). Consistent with other COVID-19 studies (e.g., Crasta et al., 2020; Chong et al., 2021), we found that veterans with higher levels of psychological flexibility reported fewer negative impacts during COVID-19 related to substance use, mental health, and pandemic-related quality of life. These findings add to the increasing evidence, with differences across populations, underlying conditions, and waves of the pandemic, that psychological flexibility is an important component of resiliency, protecting against worsening quality of life and psychological concerns within the context of COVID-19.

Notably, the positive association between psychological flexibility and study outcomes appeared strongest for veterans self-reporting a chronic pain diagnosis. Specifically, psychological flexibility demonstrated the greatest benefits for increased mental health functioning and limiting deleterious pandemic-related effects for those with a chronic pain diagnosis. This partially supported our hypothesis and is consistent with evidence that psychological flexibility is associated with increased functioning for those with chronic pain and may be protective against pandemic-specific stress (Yu et al., 2021).

Our findings also add important nuance to the apparent benefit of psychological flexibility as a protective factor, suggesting it is increasingly beneficial for veterans with more significant clinical profiles (e.g., those with both SUDs and chronic pain). This is consistent with the theory of psychological flexibility that people who are more willing to experience difficult thoughts and feelings in the service of committed action guided by their values would potentially experience less pandemic-related quality of life changes and mental health issues. Psychological flexibility may be an especially important protective factor for veterans with chronic pain, who are more likely to have multiple functional and psychological conditions that could be impacted by the pandemic. As psychological flexibility is considered a transdiagnostic mental health resiliency process, its potentially potent effects for veterans with chronic pain underscores its importance as a protective factor.

Unlike veterans with chronic pain, those without chronic pain did not show differences in the impact of COVID-19 on quality of life based on their psychological flexibility. Veterans with problematic substance use but no chronic pain reported similar levels of COVID-19 impacts on quality of life, regardless of their level of psychological flexibility. This could be because for veterans who were managing specifically problematic substance use, quality of life was less impacted by the secondary difficulties that veterans with comorbid chronic pain also cope with, such as clinic closures, physical health concerns, depression, and anxiety (Morasco et al., 2011b; Burke et al., 2015). It is also possible that veterans with chronic pain and substance use issues faced the double disadvantage of managing both substance use issues and pain during COVID-19. Specifically, these veterans may have experienced the reciprocal and negative cycle that occurs when pain is treated with recreational substances, which negatively impacts long-term pain outcomes and results in further mood issues due to the added impact of problematic substance use (Ditre et al., 2019). Higher levels of psychological flexibility may have buffered against this “double disadvantage,” as it can be particularly useful when external challenges arise that require re-evaluating values, health goals, and viable activities (Roux et al., 2022). Thus, psychological flexibility may be more strongly beneficial for quality of life for veterans actively managing chronic pain and its subsequent health issues during the pandemic. Overall, our results provide additional support for the relevance of psychological flexibility in supporting mental health functioning and quality of life (notably for veterans with co-occurring pain and problematic substance use) during the pandemic, meriting further work in this area.

### 4.1. Limitations

Several limitations to this study exist. It is important to note that this is cross-sectional data, so these results identify important associations as foundations for further research hypotheses, but should not be construed as causal findings. Additionally, as the purpose of this survey was to assess the experiences of veterans in the community during COVID-19, and thus we utilized an online, anonymous survey unlinked to medical records, we were unable to verify self-reported diagnostic information related to chronic pain (though veterans in our sample reporting a chronic pain diagnosis also demonstrated significantly higher PEG-3 scores, compared to veterans not reporting a chronic pain diagnosis). Second, our participant sample was not diverse in terms of race, ethnicity, or gender, potentially limiting generalizability. As the presence of pain and substance use comorbidities significantly affect multiple aspects of life, and this impact can differ for veterans with different racial, gender, and ethnic identities, future work in this area should include more diversity within their sample. Third, as the aim of our primary study was to assess veterans reporting problematic substance use, we did not have a comparative veteran sample with chronic pain but without self-reported substance use concerns to include in our analyses. Finally, given that we used a measure for the overall construct of psychological flexibility, the individual components of this construct (e.g., acceptance, mindfulness, valued living) could not be evaluated and represent an important area for future research.

## 5. Conclusion

Co-occurring problematic substance use and chronic pain are common, and our results provide evidence that a modifiable resiliency process (i.e., psychological flexibility) can be targeted to improve quality of life and mental health functioning. Given the transdiagnostic nature of psychological flexibility, these benefits may be generalizable to other conditions and warrant further study to explore the clinical applications of these results. Lastly, focusing on only chronic pain or substance use concerns when treating veterans with both issues may not adequately address the interrelationship between both conditions, which may be especially detrimental during moments of challenge, both personally and globally. Future research should continue to investigate the interaction between pain and substance use issues and protective factors such as psychological flexibility.

## Data availability statement

The datasets presented in this article are not readily available because owing to privacy and ethical considerations, the final data sets underlying publications resulting from this research will not be publicly shared outside of the Department of Veterans Affairs. On request and with principal investigator (EDR) permission, a deidentified, anonymized data set will be created and shared pursuant to a data use agreement, appropriately limiting the use of the data set. Requests to access the datasets should be directed to ER, Erin.Reilly@va.gov

## Ethics statement

The studies involving human participants were reviewed and approved by VA Bedford Healthcare System R&D Committee. The ethics committee waived the requirement of written informed consent for participation.

## Author contributions

All authors listed have made a substantial, direct, and intellectual contribution to the work and approved it for publication.

## Funding

This work was supported by funds from the VISN 1 New England Mental Illness Research, Education, and Clinical Center (MIRECC) for COVID-19 veteran research (PI: ER). The findings and interpretations of the data expressed in the article are the sole responsibility of the authors and do not necessarily represent the views of the Department of Veterans Affairs.

## Conflict of interest

The authors declare that the research was conducted in the absence of any commercial or financial relationships that could be construed as a potential conflict of interest.

## Publisher’s note

All claims expressed in this article are solely those of the authors and do not necessarily represent those of their affiliated organizations, or those of the publisher, the editors and the reviewers. Any product that may be evaluated in this article, or claim that may be made by its manufacturer, is not guaranteed or endorsed by the publisher.
